# A simplified method for determining earthquake early warning thresholds in high speed railways

**DOI:** 10.1038/s41598-025-17842-0

**Published:** 2025-09-25

**Authors:** Hao Li, Hongru Zhang, Hua Pan

**Affiliations:** 1 Institute of Geophysics, China Earthquake Administration, Beijing, 100081 People’s Republic of China; 2 School Civil Engineering, Beijing Jiaotong University, Beijing, 100044 People’s Republic of China

**Keywords:** High-speed railway, Shaking table test, Numerical model, Track irregularity, Earthquake early warning threshold, Superposition principle, Derailment coefficient, Civil engineering, Mechanical engineering

## Abstract

This study conducts an exploration of determining the earthquake early warning thresholds for high-speed railways. Through shaking table tests and the analysis of two-dimensional and three-dimensional numerical models, an innovative approach to deriving earthquake early warning thresholds has been proposed. Firstly, the accuracy of the two-dimensional vehicle-track numerical model was validated, by comparing with the results of shaking table tests. Secondly, this paper discusses the impact of track irregularities on the risk of train derailment, pointing out that under seismic action, the correlation between derailment risk and vehicle speed is limited, and is more related to track conditions. Furthermore, by analyzing the superimposed effects of track irregularities and seismic action, the derailment patterns of trains during earthquakes were revealed. Finally, combining the safety margin of derailment indicators and the principle of superposition effects, this paper uses six different seismic waves to calculate and analyze the two-dimensional vehicle-track model, providing the earthquake early warning threshold for high-speed railways. This method offers a new theoretical foundation and practical guidance for the design and implementation of high-speed railway earthquake early warning systems.

## Introduction

China’s high-speed rail network is rapidly expanding, with the operational mileage reaching 45,000 km by the end of 2023. Among this, the “Eight Vertical and Eight Horizontal” main corridors have been completed and put into operation for 36,400 km, accounting for 80% of the total mileage. The high-speed rail network has covered most urban areas in China. However, due to China’s location at the intersection of the Eurasian Seismic Belt and the Pacific Ring of Fire, seismic activity is frequent, with nearly half of the cities located in areas of high seismic intensity, specifically in zones of degree VII and above. Many high-speed rail lines, including the Beijing-Tianjin Intercity Railway, the Beijing-Guangzhou High-Speed Railway, and the Beijing-Shanghai High-Speed Railway, pass through regions threatened by high seismic intensity. The Peak Ground Acceleration (PGA) zoning map of China’s seismic activity^1^ shows that these areas are subject to significant seismic hazards.

**Fig. 1:**
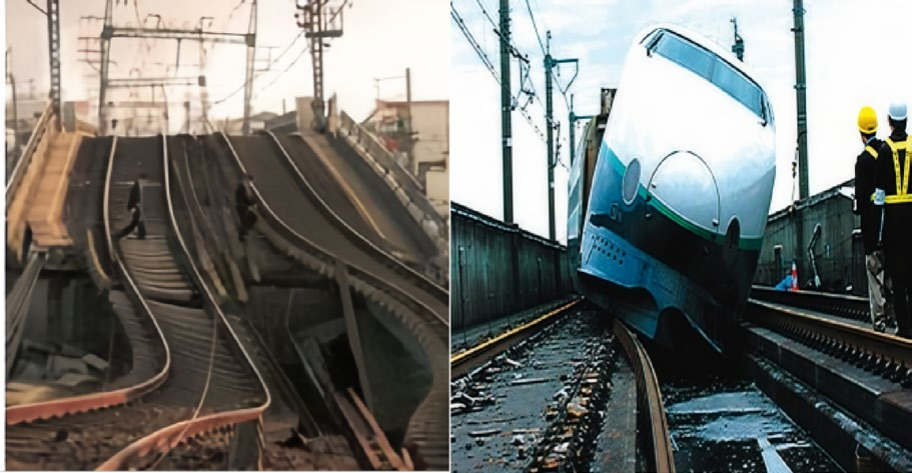
Earthquake-induced rail transit damage^4,5^.

**Fig. 2:**
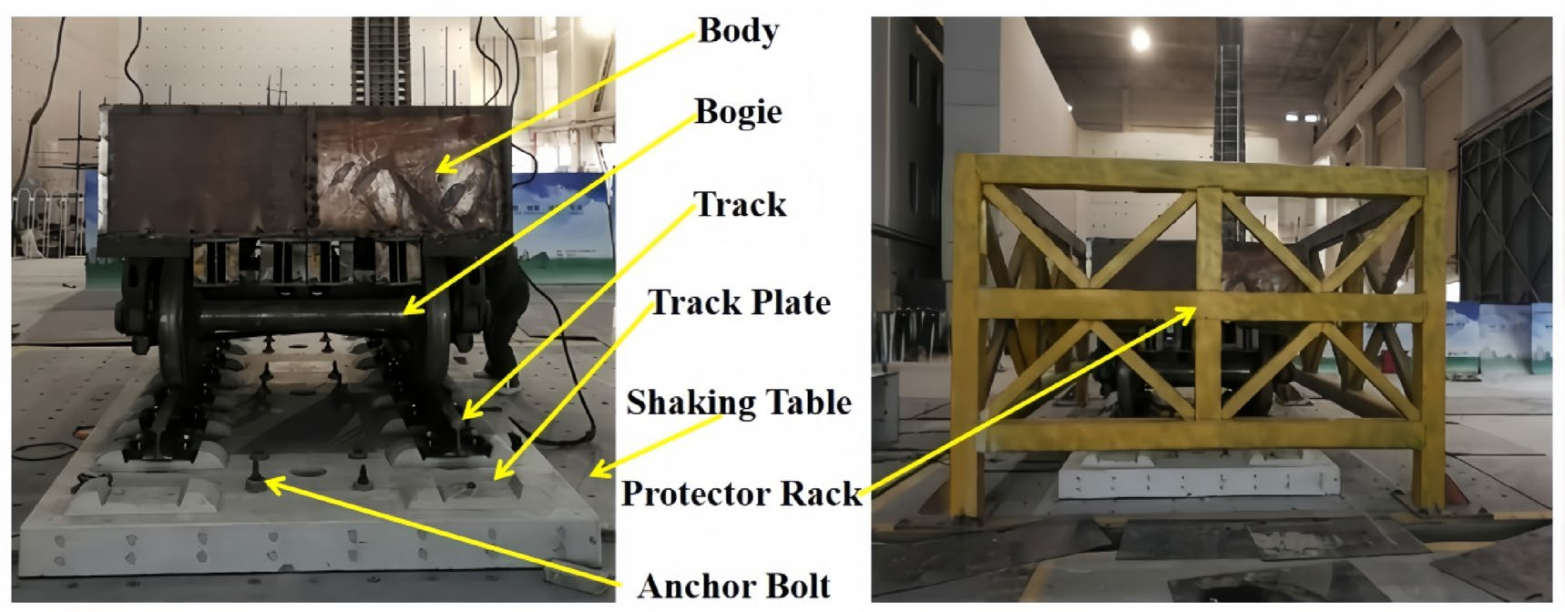
Front view and side view of the vehicle-track shaking table test.

**Fig. 3:**
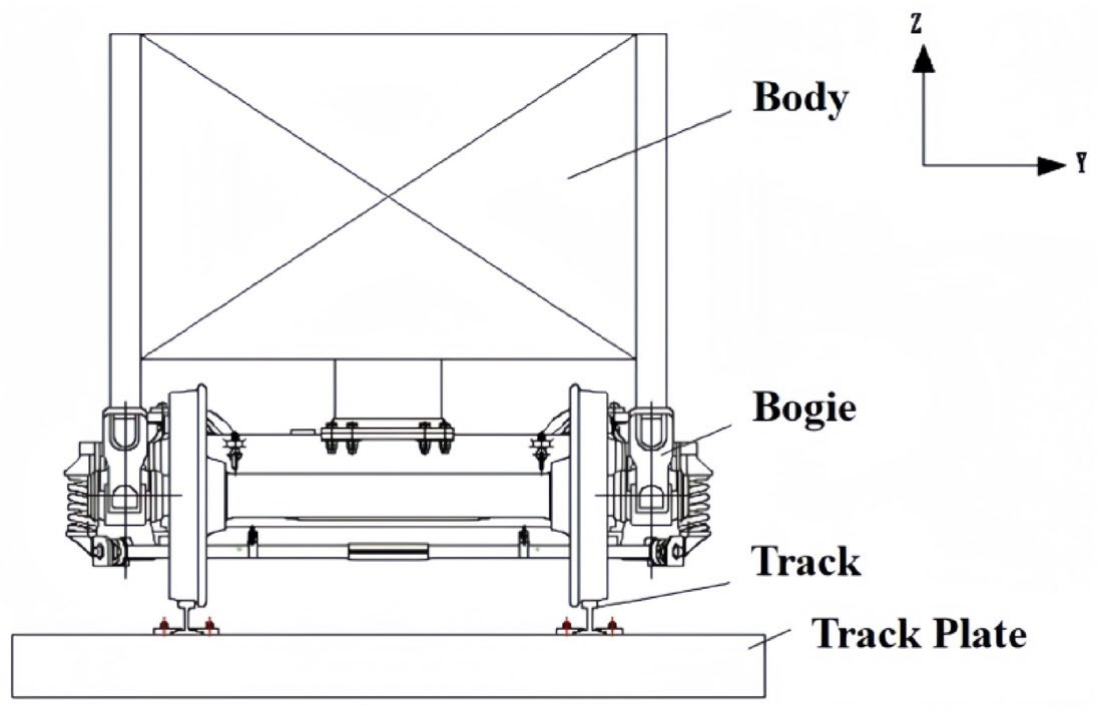
Vehicle-track model shaking table test design.

Throughout history, train accidents caused by earthquakes have not been uncommon. For instance, during the Tangshan earthquake in 1976, several passenger and freight trains derailed or overturned in areas of intensity VII and IX^2^. The 1995 Hanshin earthquake in Japan also resulted in train derailments and damage^3,4^, as shown in Fig. 1. During the 2004 Niigata earthquake in Japan, a Shinkansen bullet train derailed while operating at high speed^5,6^, as depicted in Figs. 1, 2, 3. The 2008 Wenchuan earthquake caused severe damage to the Baoji-Chengdu Railway, Chengdu-Chongqing Railway, and Chengdu-Kunming Railway, with numerous freight and passenger trains stranded and station infrastructure damaged. In 2010, the Kaohsiung earthquake in Taiwan led to the derailment of a carriage from a passenger train^7^.

To address the threat of earthquakes to the safe operation of high-speed railways, in addition to implementing seismic resistance designs, earthquake early warning systems have also become one of the key measures. Countries such as Japan have deployed seismic monitoring equipment along high-speed railway lines to achieve real-time early warnings for trains, effectively reducing the risk of derailment caused by earthquakes. Although China started relatively late in the field of high-speed railway earthquake early warning, it has begun to deploy seismic monitoring systems on some routes, laying the foundation for the establishment of an early warning system.

The practice of earthquake early warning for the Japanese Shinkansen is particularly worth emulating. In the 1960s, Japan began developing a railway earthquake early warning system. By monitoring with seismographs and combining the horizontal acceleration limit values and dynamic response amplification factors, Japan set the early warning threshold for the Shinkansen at 40 gal. The determination of this threshold is based on extensive engineering experience and strict safety standards. Other countries, such as France, have set the earthquake early warning threshold for the Mediterranean line at 65 gal^8–13^. Chinese scholars have started paying attention to the issue of high-speed railway earthquake early warning since the beginning of this century. Liu Lin et al.^14^ proposed an early warning threshold of 45 gal for the Beijing-Shanghai High-Speed Railway through seismic response analysis and calculation. Sun Li et al.^15^ introduced the energy spectral density intensity SI index, providing a new perspective for determining the early warning threshold. Liu Zhi et al.^16^, considering the excitation of track irregularities, proposed an early warning threshold related to vehicle speed based on dynamic response at different speeds. Additionally, numerous scholars^17–23^ have conducted extensive research on the safety issues of high-speed railway operations under the influence of earthquakes, and their findings have provided valuable experience and insights for the in-depth progress of this study.

The development of foreign high-speed railway earthquake early warning systems^24–34^ can be divided into three stages: threshold early warning^35^, UrEDAS system^36–39^, and earthquake detection system^40,41^. China’s research on high-speed railway earthquake early warning started relatively late and has not yet been tested by an earthquake, lacking relevant experience in emergency handling and post-earthquake train operation control braking. It can draw on foreign high-speed railway earthquake early warning processing technology, starting first with the selection of earthquake early warning thresholds, and then transitioning to other advanced warning models (such as seismic source parameter analysis during the earthquake, post-earthquake train braking control, etc.) after the technology of earthquake early warning thresholds is relatively mature and stable on high-speed railways. Therefore, in-depth analysis and research should first be conducted on the determination and selection of earthquake early warning thresholds for China’s high-speed railways.

The determination and selection of the earthquake early warning thresholds for high-speed railways are closely related to the dynamic response of the wheel-rail system and the derailment coefficients under the action of earthquakes. However, it is currently very difficult to determine the dynamic response of the wheel-rail system under the action of earthquakes through experiments or actual measurements. The main reasons are: (1) It is a rare coincidence when an earthquake occurs and there just happens to be a train passing by nearby, making it difficult to obtain actual measured data of the wheel-rail dynamic response; (2) There is currently no effective way to test the dynamic response of the wheel-rail system during the operation of the train under the action of earthquakes. Most scholars use numerical simulation methods to analyze the dynamic response of the wheel-rail system under the action of earthquakes, without comparison and verification of model tests, making it difficult to judge the correctness of the numerical simulation. The combined action of seismic loads and track irregularity excitation is the main reason affecting the dynamic response of the wheel-rail system. In view of the above challenges, in order to study the determination and selection of earthquake early warning thresholds for high-speed railways more effectively, it is recommended to discuss the impact of seismic loads and track irregularity on the dynamic response of the wheel-rail system separately. This method helps to more clearly identify and quantify the impact of various factors, providing a more solid foundation for the study of earthquake early warning thresholds.

## Research methods

In the study of earthquake early warning thresholds for high-speed railways, it is crucial to consider the interaction and motion characteristics between the vehicle and the track under the action of earthquakes. However, due to the complexity of experimental verification, especially conducting experiments on the dynamic interaction between wheels and tracks during earthquakes, is very challenging. To date, only Japan has conducted seismic response tests on full-scale vehicle-track models using a rolling vibration table^33,42–45^. In light of the lack of corresponding experimental conditions domestically, this study proposes an innovative approach: First, conduct vibration table model tests on vehicles in a non-operational state, and verify the two-dimensional numerical simulation model of the vehicle-track based on the test results; then, further construct and verify a three-dimensional numerical simulation model of the vehicle-track, which comprehensively considers track irregularities and vehicle movement states to deeply analyze the vehicle’s derailment behavior under seismic conditions. Next, by comparing the impact of track irregularities, seismic action, and their combined action on vehicle derailment indicators, the derailment laws of operating vehicles under seismic action are revealed. That is, the derailment indicators of vehicles can be predicted by the superposition of track irregularities and seismic action. Specifically, the operating state of vehicles under seismic action can be regarded as the superposition of vehicles operating on irregular tracks and vehicles operating on smooth straight tracks under seismic action. Furthermore, the latter can be approximately regarded as the state of vehicles being stationary on the track under seismic action, i.e., the vehicle-track vibration table test. Therefore, the operating state of vehicles under seismic action can be simplified as the superposition of vehicles operating on irregular tracks and vehicles being stationary on the track under seismic action.

Since the derailment indicators of vehicles operating on irregular tracks are usually within a safe range and relatively certain, this study believes that when analyzing the earthquake early warning thresholds for high-speed railways, it is possible to consider only the derailment indicators of vehicles being stationary on the track under seismic action. In this way, the simplified two-dimensional vehicle-track model verified by the vibration table can be used to determine the earthquake early warning threshold, providing a more practical and efficient analysis method for the seismic safety of high-speed railways.The Chinese “High-Speed Railway Design Code (Trial)” specifies a derailment coefficient limit of Q/P ≤ 0.8, while the International Union of Railways sets the limit at Q/P ≤ 1.2^46–49^.The derailment coefficient is defined as the ratio of the lateral force “Q” acting on the wheel to the vertical force “P” acting on the wheel, which is expressed as Q/P. This coefficient is a critical indicator used to assess the risk of derailment under seismic conditions.

The specific research methods are introduced as follows:

1. Conduct related research and testing through full-scale vehicle-track model vibration table tests under different indoor seismic load conditions, analyze and discuss the dynamic response of the vehicle-track model and its derailment mechanism under seismic action, and provide a theoretical basis for the study of vehicle earthquake early warning thresholds.
2. According to the vehicle-track dynamics theory, establish a two-dimensional vehicle-track dynamics numerical model, and the parameters of the entire calculation model are consistent with the vibration table model test parameters. Compare the above test results with the calculation results of the vehicle-track numerical calculation model to verify the correctness of the vehicle-track numerical calculation model.
3. Establish a three-dimensional vehicle-track dynamics numerical model considering track irregularities, and through the comparative analysis of the impact of track irregularities, seismic action, and their combined action on the earthquake condition vehicle derailment indicators, study the derailment laws of operating vehicles under seismic action, and verify that the derailment indicators of vehicles under seismic action can be obtained by the superposition of track irregularities and seismic action. Then, the operating state of vehicles under seismic action can be regarded as the superposition of vehicles operating on irregular tracks and vehicles operating on straight tracks under seismic action. The latter can be approximated as the state of vehicles being stationary on the track under seismic action, i.e., the vehicle-track vibration table test. Therefore, the operating state of vehicles under seismic action can be simplified as the superposition of vehicles operating on irregular tracks and vehicles being stationary on the track under seismic action.
4. Since the derailment indicators of vehicles operating on irregular tracks are usually within a safe range and relatively certain, when analyzing the earthquake early warning thresholds for high-speed railways, it is possible to consider only the derailment indicators of vehicles being stationary on the track under seismic action. In this way, the simplified two-dimensional vehicle-track model verified by the vibration table can be used to determine the earthquake early warning threshold, providing a more practical and efficient analysis method for the seismic safety of high-speed railways.
5. It is planned to propose preliminary suggestions for determining the earthquake early warning threshold for high-speed railways on the basis of two-dimensional vehicle-track numerical simulation. By inputting six different horizontal seismic waves, conduct seismic response analysis on the vehicle-track model, and using the above superposition principle and the safety redundancy of derailment indicators, provide the early warning thresholds corresponding to different derailment coefficient limits and different wheel load reduction rate limits under the action of seismic forces. Provide a certain reference value for the practical engineering application of high-speed railways.

## Vibration table test and numerical model verification

### Test overview

The experiment was conducted in the seismic resistance laboratory of the China Academy of Building Research. The components of the test model, from top to bottom, are the car body, bogie, steel rail, clip, track slab, and vibration table. A schematic diagram of the vibration table model is shown in Fig. 2. The carriage is made by welding angle steel and steel plates, and the car body base adopts a grid box structure, which is composed of longitudinal and transverse rectangular steel tubes welded together. The car body and bogie are connected by welding on both sides of the side bracing and the center plate. To better simulate the mass of half a car body, the method of adding counterweights is used for mass simulation. The track slab is our country’s independently developed CRTS Ⅲ type track slab, the bogie adopts the domestically produced K2 type bogie from Shijiazhuang Vehicle Factory, the steel rail is a 60 kg/m rail, and the clip adopts the WJ-8b type clip. The surface of the track slab is designed with holes, and 14 steel bolts with a diameter of 30 mm are used to anchor the track slab in the center of the vibration table, allowing the vehicle-track model to vibrate synchronously with the vibration table. The size parameters of the vehicle track model are shown in Table1.

**Table 1:** Main performance parameters of vehicle vibration table test components.

No	Main parameters	Unit	Parameter value
1	Car-body weight	t	2
2	Car-body counter weight	t	14
3	Bogieweight	t	4
4	Track plate weight	t	6.3
5	Track plate length	mm	4980
6	Track plate width	mm	2500
7	Track plate thickness	mm	200
8	Gauge	mm	1435
9	Car body width	mm	2400
10	Car body length	mm	2900
11	Car body base thickness	mm	250
12	Wheel diameter	mm	840

### Loading conditions

The vehicle-track model was subjected to various seismic waves, including five-cycle sine waves, El-centro waves, Kobe waves, and artificial waves. Each seismic wave was applied in a graded manner. There were a total of 24 scenarios with seismic wave inputs in the horizontal direction and one scenario with bidirectional inputs, where both horizontal and vertical El-centro waves were applied simultaneously, totaling 25 scenarios. The directions of seismic wave application were the Y-direction and the Z-direction, with the Y-direction being horizontal and the Z-direction being vertical. A simplified diagram of the test model design is shown in Fig. 3. Due to the lengthy nature of the test results and analysis, this paper does not provide a complete display. The focus of this study is to utilize these test results to compare and verify the developed two-dimensional vehicle-track numerical model, thereby providing reliable theoretical support and technical support for the derivation of earthquake early warning thresholds for high-speed railways.

### Verification of the two-dimensional vehicle-track numerical model

**Fig. 4:**
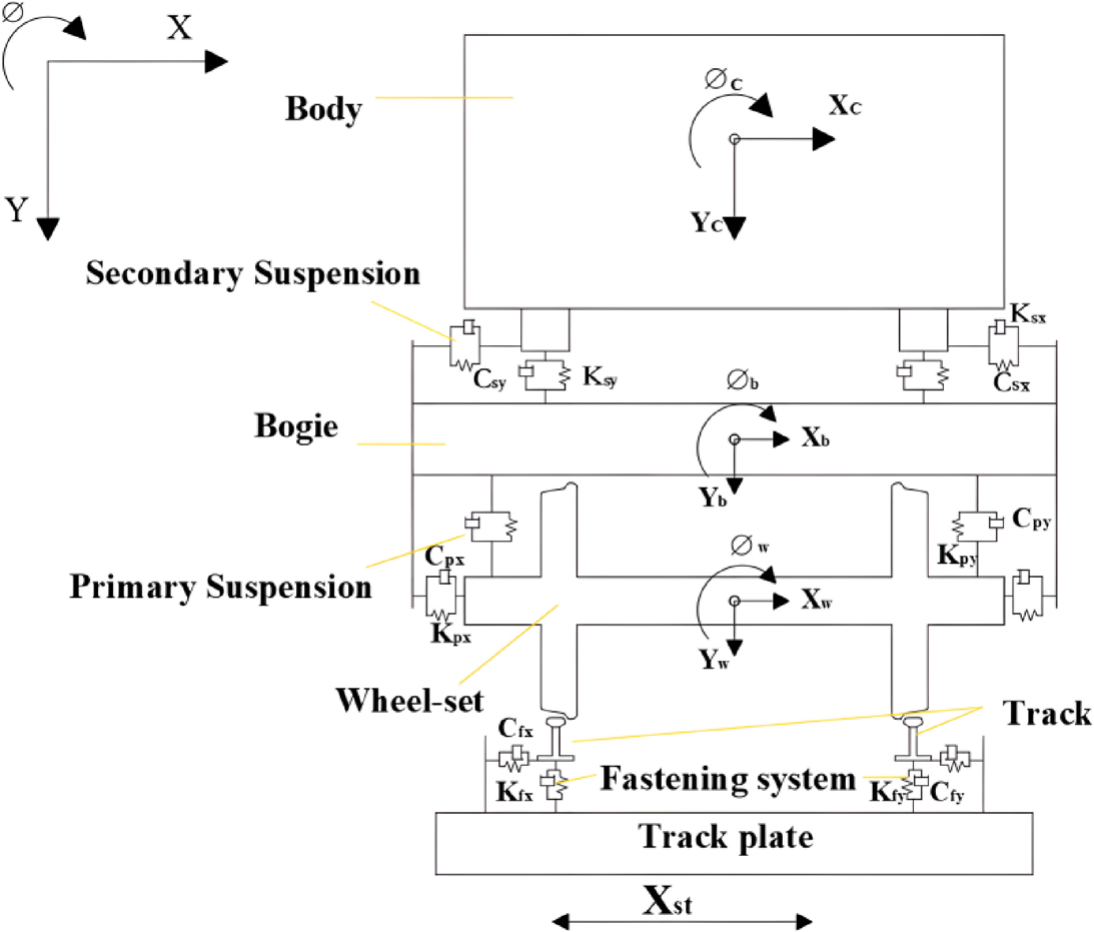
Vehicle-track dynamics model.

Based on the principles of vehicle-track coupling dynamics^50^, a two-dimensional vehicle-track model was established using the ABAQUS finite element software. The model, from top to bottom, consists of the car body, bogie, wheel set, steel rail, fastener, and track slab. According to vehicle-track dynamics theory, a high-speed train car can be simplified to consist of a car body, frame, wheel set, and primary and secondary springs. The wheel set and frame are connected by primary springs, while the car body and frame are connected by secondary springs. Spring damping elements are established between the track and the track slab to simulate the fasteners. The entire model calculation schematic is shown in Fig. 4.

**Fig. 5:**
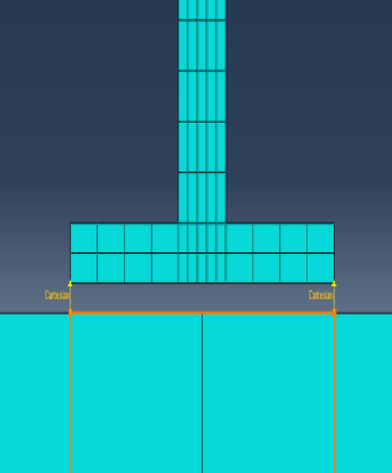
Schematic diagram of fastener spring unit (Fig. 5 were generated using^51^).

In the vehicle model, both the primary and secondary springs, as well as the fastener models, are simulated using Cartesian spring elements. The Cartesian spring elements^51^ are unconstrained relative motion components, and the available relative motion components are U1 (the horizontal direction component). The local coordinate system for the first endpoint is required to be optional (users can choose whether to define a local coordinate system at this endpoint), and the local coordinate system for the second endpoint is required to be Ignored (users do not need to define a local coordinate system at this endpoint). Figure 5 illustrates the schematic of the fastener spring element in the vehicle-track model. The dynamic parameters of each component of the upper structure of the track are presented in Table 2.

**Table 2:** Track upper structure parameters^50^.

Name	Parameter	Symbol	Unit	Value
Vehicle body	Mass	*M*_c_	kg	15,030
	Moment of inertia about the z-axis	*I*_cz_	kg m^2^	7.506 × 10^4^
Bogie	Mass	*M*_b_	kg	3890
	Moment of inertia about the z-axis	*I*_bz_	kg m^2^	2260
Wheel-set	Mass	*M*_w_	kg	1755
	Moment of inertia about the z-axis	*I*_wz_	kg m^2^	915
First series spring	Lateral stiffness	*K*_px_	kN/m	12,000
	Vertical stiffness	*K*_py_	kN/m	1260
	Vertical damping	*C*_pz_	kN s/m	6
Second series spring	Lateral stiffness	*K*_sx_	kN/m	176
	Vertical stiffness	*K*_sy_	kN/m	235
	Lateral damping	*C*_sx_	kN s/m	1.22
	Vertical damping	*C*_sy_	kN s/m	16.5

**Fig. 6:**
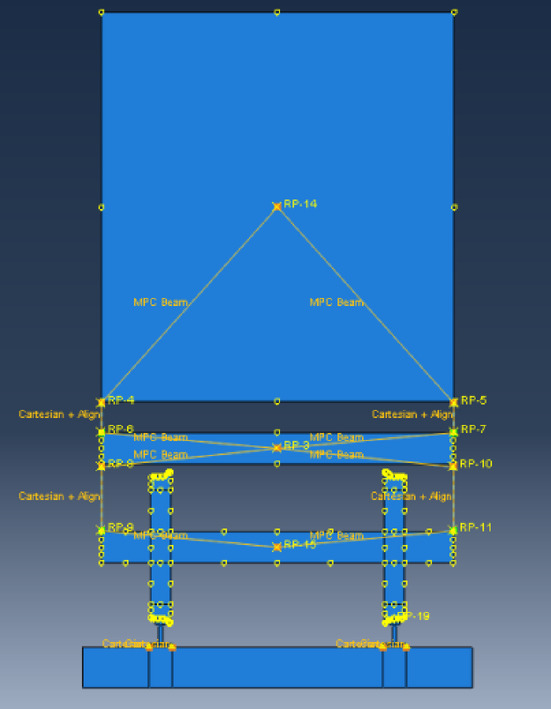
Vehicle track dynamics model (Fig. 6 were generated using^51^).

In the model, the gauge of the tracks is set to 1435mm according to the specifications. The track slabs are laid beneath the steel rails and are constructed from concrete. The tracks, track slabs, and fastener models all adopt a linear elastic constitutive model, with their dynamic and geometric parameters detailed in Tables 3 and 4, respectively. The entire vehicle-track dynamic numerical model is depicted in Fig. 6.

**Table 3:** Track system dynamic parameters.

Name	Thickness (m)	Elasticity modulus GPa	Poisson ratio	Density kg/m^3^
Steel rail	0.176	206	0.3	7800
Track slab	0.19	35.5	0.1	2400

Fastener stiffness 78kN/mm damping coefficient 50 kN s/m.

**Table 4:** Track system geometrical parameters.

Name	Position	Unit	Value
Wheel	Inner diameter	mm	845
	External diameter	mm	915
	Thickness	mm	145
Track cross section	High	mm	176
	Bottom width	mm	150
	Top width	mm	73
	Waist depth	mm	16.5
Track slab	Width	mm	2500
	Thickness	mm	260

#### Wheel-rail contact setup

**Fig. 7:**
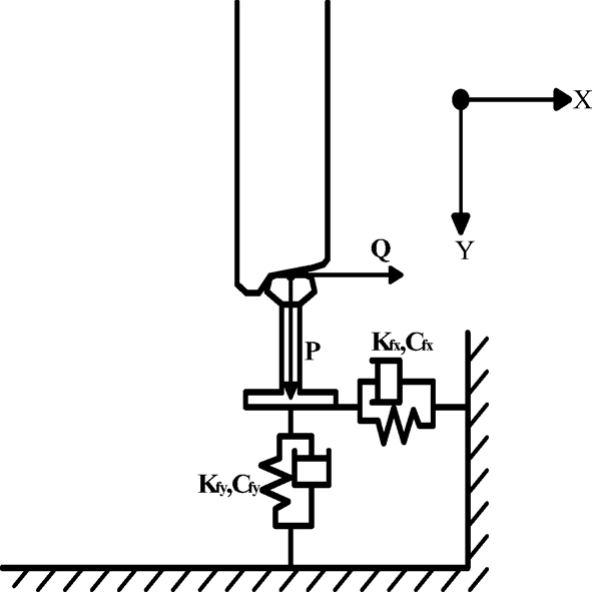
Contact diagram of wheel and rail.

In the vehicle-track dynamics model, the interaction between the wheel and rail is simulated by applying contact pairs. The spatial dynamic contact force model^52–55^ consists of two parts: the tangential force calculation model and the normal force calculation model for wheel-rail interaction. The tangential force employs the “Penalty contact method” for computation, with the friction coefficient of the contact surface set to 0.3. The normal force represents the relationship between the normal load and local deformation at the wheel-rail contact. The “Hertz” contact method is used for the calculation and analysis of the model. A schematic of the wheel-rail contact model setup is shown in Fig. 7.

#### Numerical model validation

The parameters of the aforementioned vehicle-track model are consistent with those of the vibration table model test. The accuracy of the two-dimensional vehicle-track computational model established in this paper will be verified by comparing it with the results of the vibration table model test.

A sine wave earthquake is applied to the bottom of the vehicle-track dynamics model to perform a seismic time-history analysis. The dynamic response patterns of wheel lift, horizontal wheel-rail contact force, and vertical wheel-rail contact force as a function of the seismic excitation time will be plotted. These results will then be compared and analyzed against the outcomes of the vehicle-track model vibration table test.

**Fig. 8:**
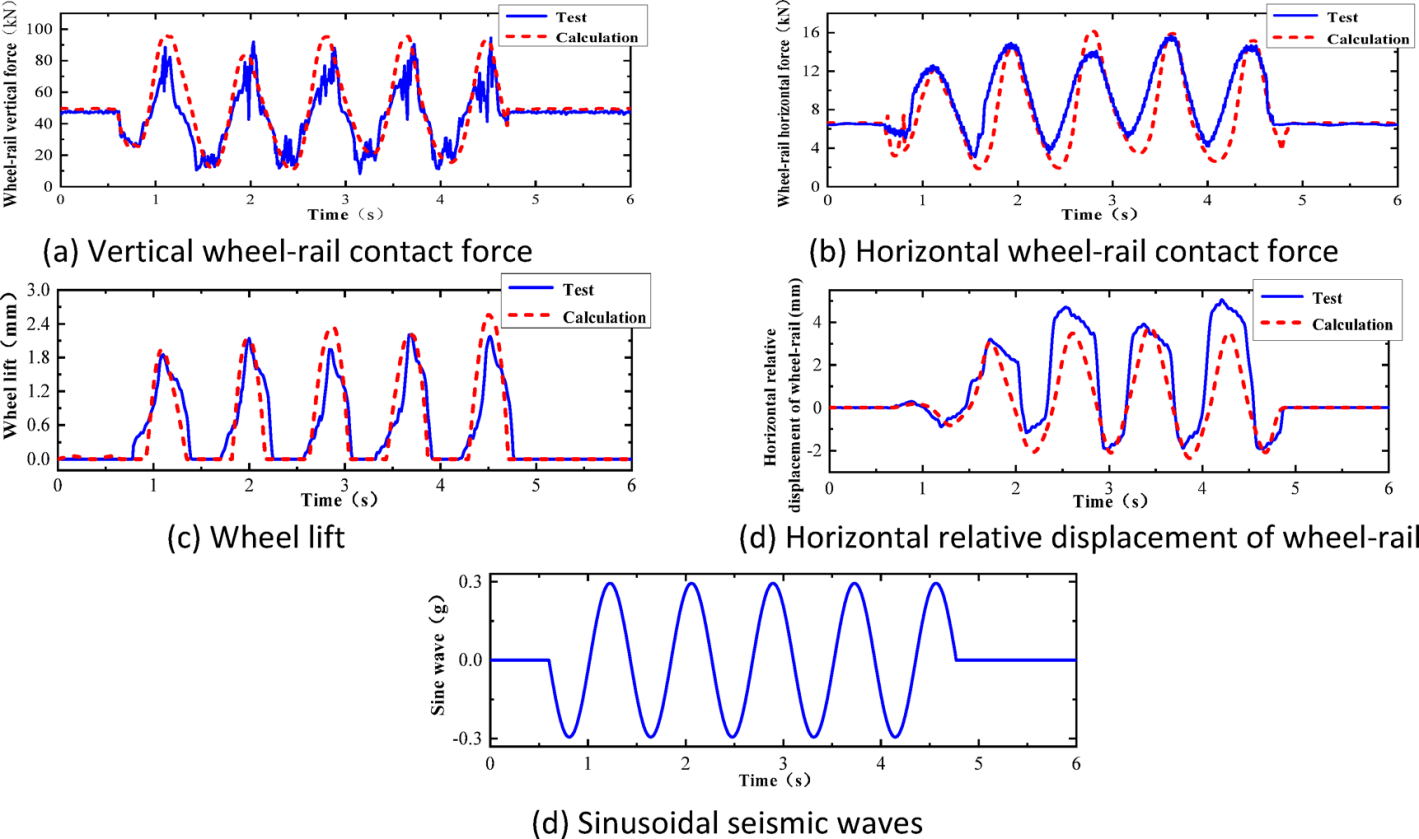
Comparison of numerical simulation results and shaking table test results.

After comparative analysis, it was found that, as shown in Fig. 8, the results of numerical simulation are in good agreement with the data from the vibration table model test in terms of overall trends. Although there are some minor differences at the detail level, these discrepancies are mainly due to the simplified nature of the numerical model, whereas the vibration table test encompasses more complex actual conditions. However, when focusing on the periodic characteristics and peak responses, it is evident that the numerical simulation results are highly consistent with the test results. This finding strongly confirms that the established two-dimensional vehicle-track numerical calculation model has a high degree of accuracy and effectiveness, thereby providing a solid foundation for further research and application.

## Validation of the three-dimensional vehicle-track model

The vehicle model consists of one car body, two bogies, and four wheel sets, making up a total of seven rigid bodies. The bogies and car body are connected by secondary springs, while the bogies and wheel sets are connected by primary springs. Each rigid body has five degrees of freedom, which are transverse movement, vertical movement, roll, pitch, and yaw. Thus, the entire three-dimensional vehicle model system has 35 degrees of freedom. Additionally, both the primary and secondary springs and the fastener models are simulated using Pro Cartesian spring elements. The parameters of the vehicle model are presented in Table 5.

**Table 5:** Track upper structure parameters.

Name	Parameter	Symbol	Unit	Value
Vehicle body	Mass	*M*_*c*_	kg	30,060
	Moment of inertia about the z-axis	*I*_*cz*_	kg m^2^	7.506 × 10^4^
	Moment of inertia about the x-axis	*I*_*cx*_	kg m^2^	2.277 × 10^6^
	Moment of inertia about the y-axis	*I*_*cy*_	kg m^2^	2.086 × 10^6^
Frame	Mass	*M*_*b*_	kg	3890
	Moment of inertia about the z-axis	*I*_*bz*_	kg m^2^	2260
	Moment of inertia about the x-axis	*I*_*bx*_	kg m^2^	2710
	Moment of inertia about the y-axis	*I*_*by*_	kg m^2^	3160
Wheel-set	Mass	*M*_*w*_	kg	1755
	Moment of inertia about the z-axis	*I*_*wz*_	kg m^2^	915
	Moment of inertia about the x-axis	*I*_*wx*_	kg m^2^	140
	Moment of inertia about the y-axis	*I*_*wy*_	kg m^2^	915
First series spring	Lateral stiffness	*K*_*px*_	kN/m	12,000
	Vertical stiffness	*K*_*py*_	kN/m	1260
	Longitudinal stiffness	*K*_*pz*_	kN/m	10,000
	Vertical damping	*C*_*pz*_	kN s/m	6
Second series spring	Lateral stiffness	*K*_*sx*_	kN/m	176
	Vertical stiffness	*K*_*sy*_	kN/m	235
	Longitudinal stiffness	*K*_*sz*_	kN/m	150
	Lateral damping	*C*_*sx*_	kN s/m	1.22
	Vertical damping	*C*_*sy*_	kN s/m	16.5

**Fig. 9:**
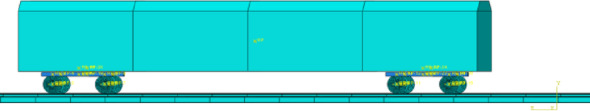
Side view of the finite element vehicle-track model (Fig. 9 were generated using^51^).

In the model, the interaction between the wheel and rail is simulated by applying contact pairs. The spatial dynamic contact force model for wheel-rail interaction consists of two parts: the tangential force calculation model and the normal force calculation model. The tangential force employs the “penalty function” method for computation, with the friction coefficient of the contact surface set to 0.3. The normal force continues to use the “Hertz” contact method for analysis of the model. The method of seismic wave input is the same as described previously, and a schematic diagram of the three-dimensional model is shown in the Fig. 9.

In the dynamic model, the inner diameter of the wheel is 845 mm, the outer diameter is 915 mm, and the thickness is 145 mm. The steel rail adopts the standard 60 rail, with a rail height of 176 mm, a lower width of 150 mm, an upper width of 73 mm, and a web thickness of 16.5 mm. The gauge of the steel rails is taken as 1435 mm according to specifications, the distance between the wheels on the same axle is 100 mm, the length of the track is 2000 m, the length of the track slab is 5 m, with a total of 400 track slabs. “Tie” constraints are applied between the track slabs, and the track slabs are laid under the steel rails as a concrete structure. Both the steel rails, track slabs, and fastener models adopt a linear elastic constitutive model, as seen in Table 3.

### Model analysis and validation

**Fig. 10:**
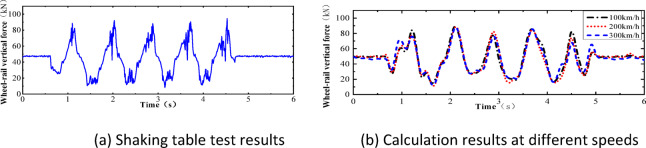
Comparison of wheel-track vertical forces between test results and calculation results.

**Fig. 11:**
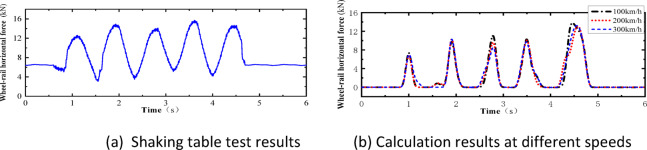
Comparison of horizontal wheel-track forces between test results and calculation results.

**Fig. 12:**
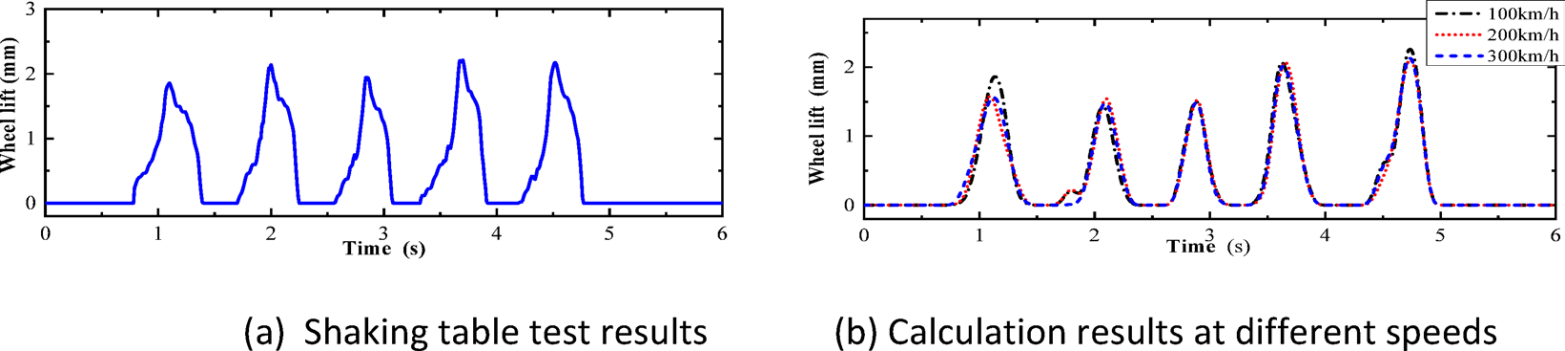
Comparison of wheel lift between test results and calculation results.

**Fig. 13:**
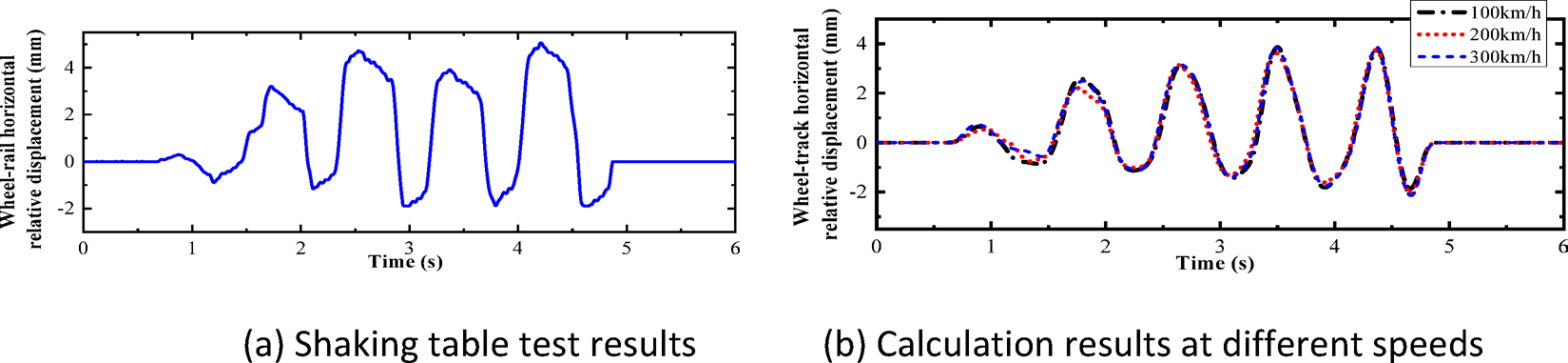
Comparison of horizontal relative displacements between test results and calculation results.

**Fig. 14:**
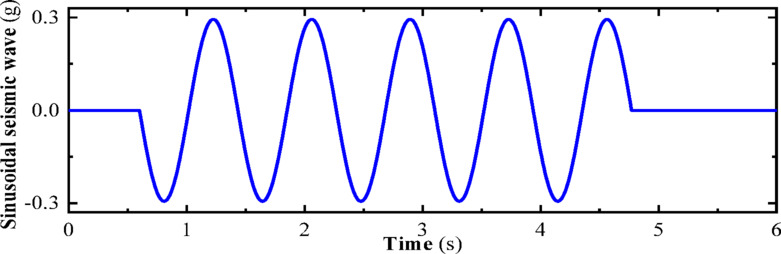
Sinusoidal seismic waves.

In this section, a sinusoidal acceleration seismic wave with a duration of five cycles and an amplitude of 0.3 g is used as the seismic input, as illustrated in Fig. 22. The vertical force between the wheel and rail, horizontal force between the wheel and rail, wheel lift amount, and horizontal displacement between the wheel and rail of the train are calculated under different train speeds. The track irregularities are not considered. The train speeds considered are 100 km/h, 200 km/h, and 300 km/h. The calculated results are compared with the results of the vibration table experiments mentioned above, as shown in Figs. 10, 11, 12, 13, 14.

As shown in Figs. 10, 11, 12, 13, 14, under the seismic action and in the absence of track irregularities, the curves of vertical force between the wheel and rail, horizontal force between the wheel and rail, wheel lift amount, and horizontal relative displacement between the wheel and rail exhibit similar trends and nearly overlap when the train travels at speeds of 100 km/h, 200 km/h, and 300 km/h. This result indicates that the influence of train speed on the dynamic response of the wheel-rail system is relatively small under seismic action when track irregularities are not considered.

Furthermore, the numerical simulation results are compared with the results of static vehicle-track model vibration table experiments. Although there are slight differences in detail, the peak values and overall waveforms of the two sets of results are basically consistent. This further demonstrates that the dynamic response of the wheel-rail system when the train is stationary under seismic action is close to that when the train travels at a constant speed on a track without considering track irregularities. This not only validates the correctness of the numerical model but also provides strong support and reference for the numerical simulation considering track irregularities in the next section.

### Establishment of the track irregularity model

**Fig. 15:**
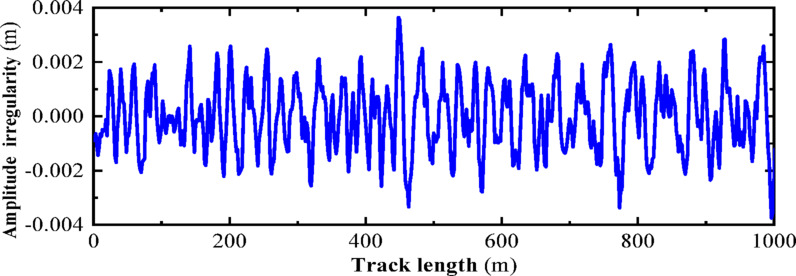
Track irregularities.

**Fig. 16:**
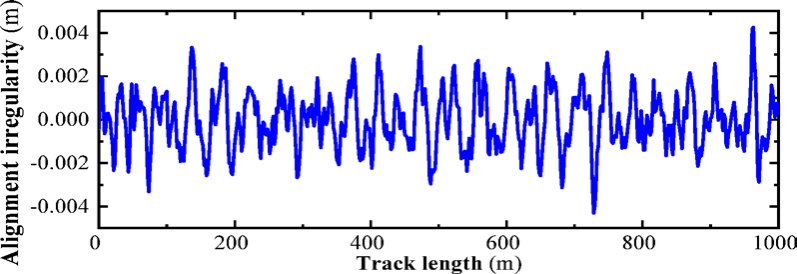
Irregular track direction spectrum.

The American track spectrum is widely applied in the study of high-speed railways in China. In this section, the inverse Fourier transform method is used to numerically simulate the American track irregularity. Based on the power spectral density function of the American track irregularity, the wavelength range of the track irregularity is set from 2 to 50 m. MATLAB programming is utilized to plot the American Level 6 track spectrum, as shown in Figs. 15 and 16, which represent the vertical and directional irregularity samples, respectively. According to the numerical simulation results, the vertical irregularity amplitude range of the American Level 6 track is [− 4, 4] (units: mm); the directional irregularity amplitude range is also [− 4, 4] (units: mm).

**Fig. 17:**
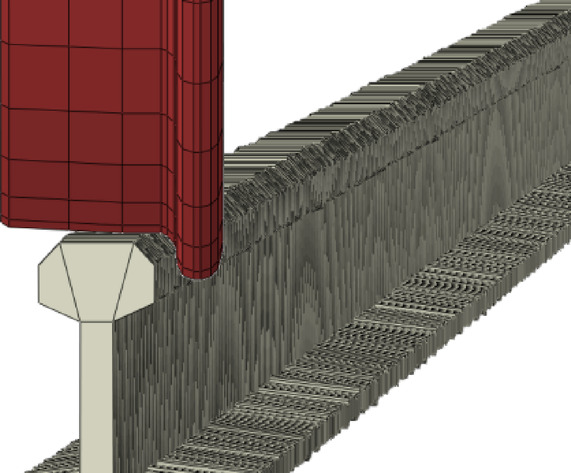
Details of track irregularities zoom in (Fig. 17 were generated using^51^).

In the software’s mesh module, the grid density of the track is used to determine the accuracy of the mileage. The track irregularity status is then achieved by modifying the coordinate positions of the track elements. Due to the large number of track elements, a programming approach is adopted to apply the track irregularity spectra in both vertical and directional orientations to the three-dimensional vehicle-track model. The figure of the track model after the addition is shown in Fig. 17.

### Analysis of wheel-rail dynamic response considering track irregularity

**Fig. 18:**
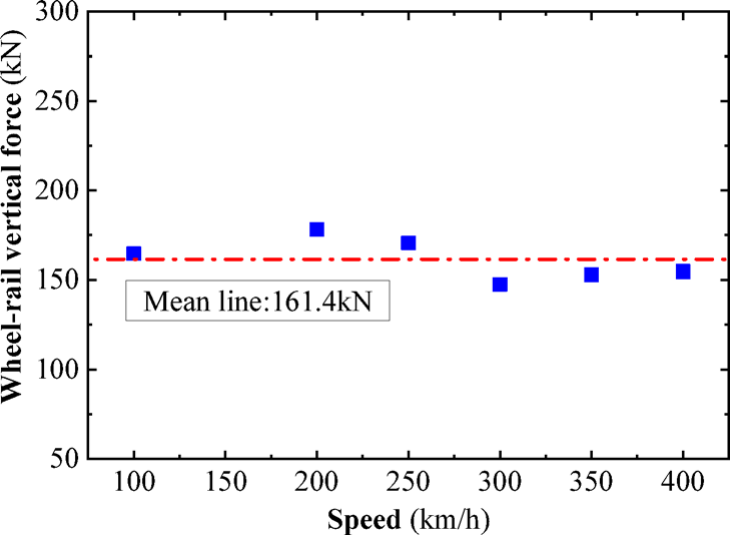
Peak response of wheel-rail vertical force.

**Fig. 19:**
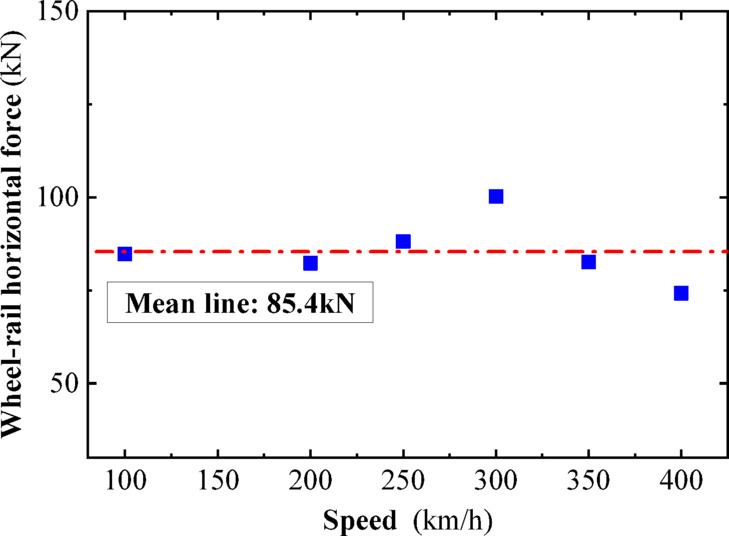
Peak response of wheel-rail horizontal force.

**Fig. 20:**
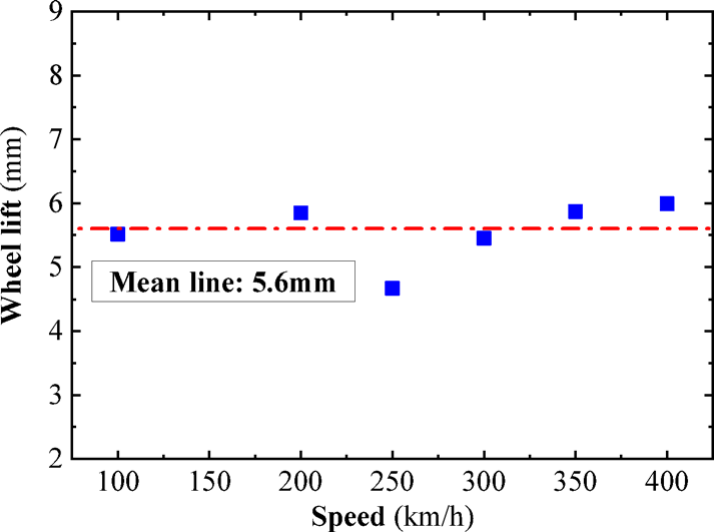
Peak response of wheel lift.

**Fig. 21:**
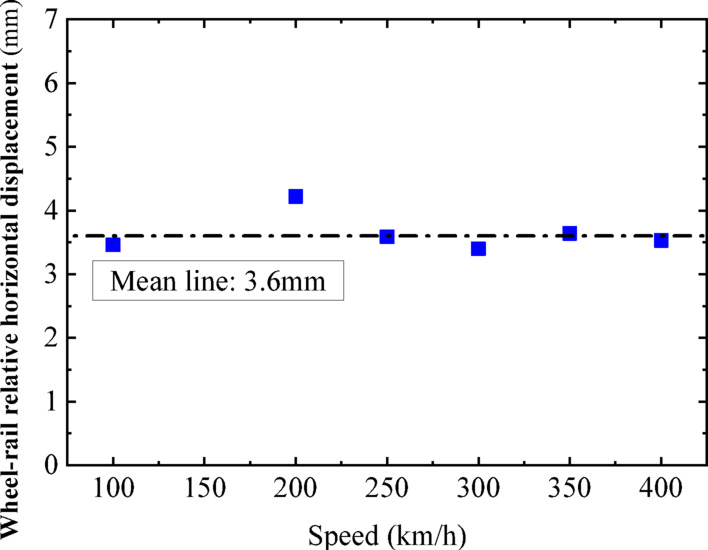
Peak response of wheel-rail relative horizontal displacement.

To further investigate the relationship between vehicle speed and wheel-rail dynamic response under the action of seismic forces after the introduction of track irregularities, sinusoidal acceleration seismic waves continue to be used as the input for ground motion. The vertical wheel-rail force, horizontal wheel-rail force, wheel lift, and horizontal wheel-rail displacement of the vehicle at different speeds after the introduction of track irregularities are calculated. The vehicle speeds considered are 100 km/h, 200 km/h, 250 km/h, 300 km/h, 350 km/h, and 400 km/h. Due to the similar dynamic response patterns of the four wheel sets, and for the sake of brevity, the peak dynamic response curves of the vertical wheel-rail force, horizontal wheel-rail force, wheel lift, and horizontal wheel-rail displacement for one set of wheels are depicted in Figs. 18, 19, 20, 21. It can be observed that when considering track irregularities, under the action of an earthquake, the impact of different vehicle speeds on the wheel-rail dynamic response is not very pronounced.

From Figs. 18, 19, 20, 21, it can be observed that as the train speed increases, the peak response of the wheel-rail vertical force does not grow linearly but fluctuates within a range of (150–175 kN). Notably, at a train speed of 300 km/h, the corresponding peak response of the wheel-rail vertical force is the smallest, at 150 kN. The peak response of the wheel-rail horizontal force fluctuates within a range of (75–100 kN), with the largest peak response occurring at a train speed of 300 km/h, reaching 100 kN. The peak response of wheel lift fluctuates within a range of (4.5–6 mm), with the smallest peak response corresponding to a train speed of 250 km/h, at 4.7 mm. The peak response of the relative horizontal displacement between the wheel and rail fluctuates within a smaller range of (3.4–4.2 mm).

Overall, when the peak acceleration of the seismic wave is 0.3 g, the wavelength range of track irregularity is (0.5 to 80 m), and the vehicle speed range is (100–400 km/h), the impact of vehicle speed on the peak values of various wheel-rail dynamic responses is not significant, and there is no clear linear growth trend. Therefore, it can be inferred that within a certain range, the derailment of vehicles under seismic action is not significantly related to vehicle speed.

Considering the operating state of vehicles under seismic action as a superposition of vehicles operating on irregular tracks and vehicles operating on smooth tracks under seismic action, the wheel-rail forces when the vehicle operates on a smooth straight track can be approximately equivalent to the wheel-rail forces when the vehicle is stationary. The dynamic response of the stationary vehicle-track under seismic action can be verified through a shaking table test, as mentioned earlier. The main drawback is that in reality, vehicle operation and seismic action may have a coupling effect. However, the aforementioned research analysis indicates that derailment under seismic action is not significantly related to vehicle speed. Therefore, the aforementioned approach can be used as a preliminary approximation to determine the seismic early warning threshold, providing some theoretical support for subsequent research on seismic early warning thresholds for high-speed railways.

## Analysis of derailment mechanism of operating vehicles under earthquake action and verification of superposition principle

Since the combined action of seismic loads and track irregularity excitation is the main reason affecting the dynamic response of wheel-rail systems, the previous sections have studied the impact of seismic loads on the dynamic response of wheel-rail systems through model vibration table tests and numerical simulations, as well as the impact of track irregularities on the dynamic response of wheel-rail systems. To further explore the internal connections between the dynamic responses of wheel-rail systems under the action of seismic loads and track irregularity excitation, this section defines the vehicle’s three states as State A, State B, and State C, which are specifically described as follows:

1. State A: The state where the vehicle runs on the track at a certain speed considering both seismic loads and track irregularities.
2. State B: The state where the vehicle runs on the track at a certain speed without seismic loads but considering track irregularities.
3. State C: The state where the vehicle is stationary on the track under the action of seismic loads.

**Fig. 22:**
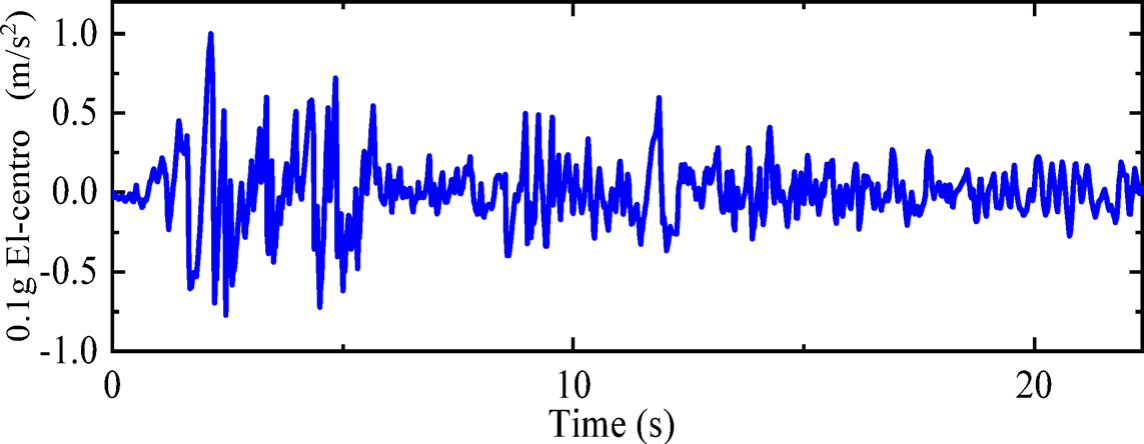
0.1 g El-Centro earthquake wave.

To analyze the derailment coefficients and wheel load reduction rates for these three states, numerical models of vehicle-track dynamics that correspond to “State A,” “State B,” and “State C” are established. The seismic wave used adopts the El-Centro earthquake wave with a peak acceleration of 0.1 g, as shown in Fig. 22.

By establishing and analyzing these models, we can better understand the derailment mechanism of operating vehicles under earthquake action and verify the superposition principle, which states that the dynamic response of vehicles under seismic loads and track irregularities can be analyzed and predicted by considering the effects of each factor separately and then combining them. This approach is crucial for developing effective seismic design and early warning strategies for high-speed railways to ensure operational safety during seismic events.

**Fig. 23:**
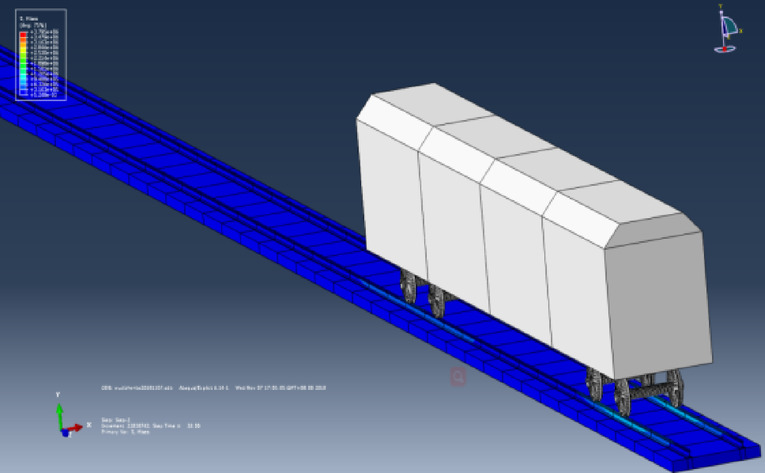
Calculation cloud drawing of state B (Fig. 23 were generated using^50^).

**Fig. 24:**
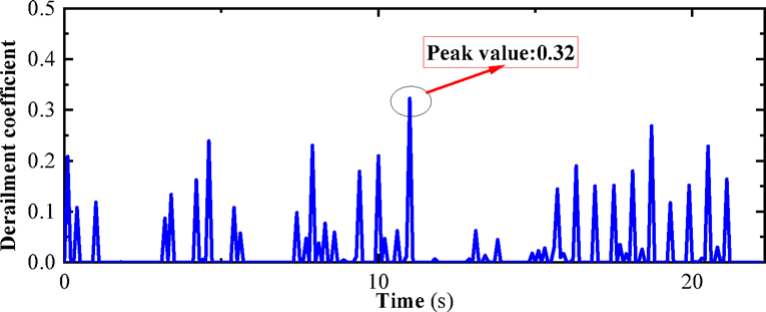
Derailment coefficient of state B.

**Fig. 25:**
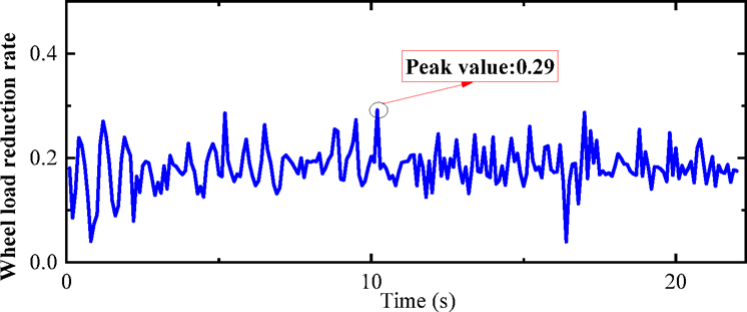
Weight reduction rate of wheel of state B.

“State B” represents the operating condition of a vehicle on the track considering track irregularities but without seismic loads. The dynamic numerical model utilized is a three-dimensional vehicle-track dynamics model that takes into account track irregularities. The speed is set at 300 km/h, and the operating time is 27 s, with no seismic loads applied. Other parameters of the model are detailed in Chapter 4. The computational cloud diagram is shown in Fig. 23: The derailment coefficient and wheel load reduction rate for “State B” (under the action of track irregularity excitation only) are calculated and summarized in Figs. 24 and 25, respectively.

**Fig. 26:**
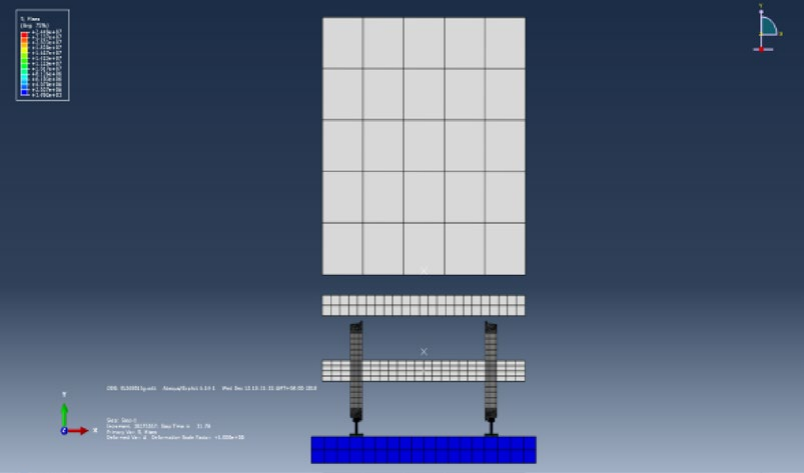
Calculation cloud drawing of state C (Fig. 26 were generated using^51^).

**Fig. 27:**
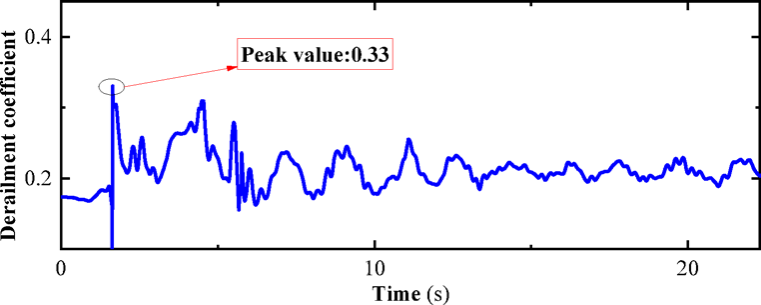
Derailment coefficient of state C.

**Fig. 28:**
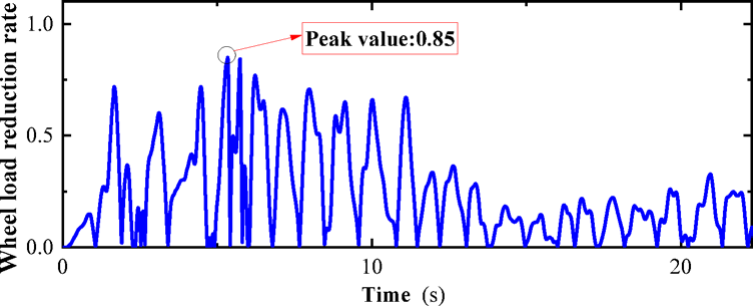
Weight reduction rate of wheel of state C.

“State C” corresponds to the condition where the vehicle is stationary on the track under the action of an earthquake. The dynamic numerical model employed is a two-dimensional vehicle-track dynamics model. An El-Centro wave with a peak acceleration of 0.1 g is applied at the bottom of the track slab in the model. Additional parameters of the model are elaborated in Chapter 3. The computational cloud diagram for the model is depicted in Fig. 26. The calculation results for the derailment coefficient and wheel load reduction rate for “State C” (under the action of seismic loads only) are statistically compiled and illustrated in Figs. 27 and 28, respectively.

**Fig. 29:**
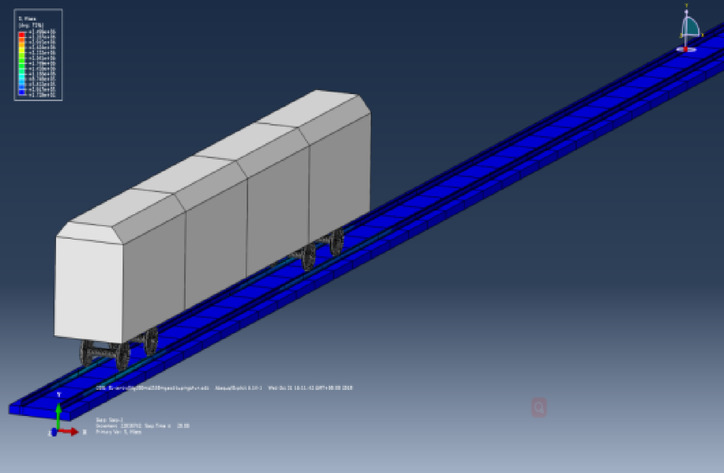
Calculation cloud drawing of state A (Fig. 29 were generated using^51^).

**Fig. 30:**
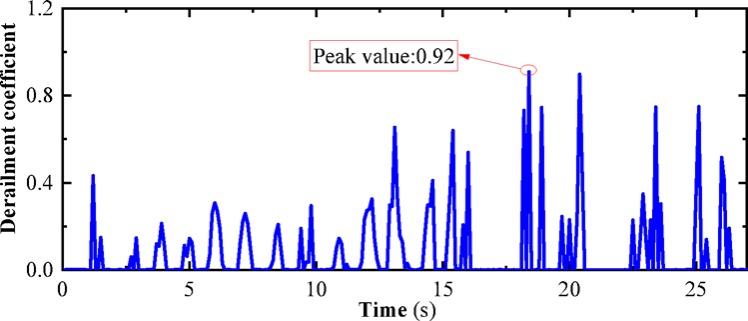
Derailment coefficient of state A.

**Fig. 31:**
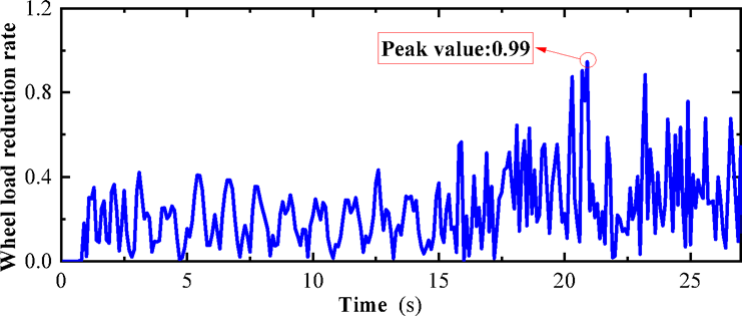
Weight reduction rate of wheel of state A.

“State A” represents the operating condition of a vehicle on the track considering both track irregularities and the effects of an earthquake. The dynamic numerical model used is a three-dimensional vehicle-track dynamics model that takes into account track irregularities. The speed is set at 300 km/h, and the operating time is 27 s. An El-Centro wave with a peak acceleration of 0.1 g is applied at the bottom of the track slab in the model. For further details on the model parameters, please refer to Chapter 4. The computational cloud diagram for the model is shown in Fig. 29. The calculation results for the derailment coefficient and wheel load reduction rate for “State A” (considering the combined effects of track irregularities and earthquake loads) are statistically compiled and presented in Figs. 30 and 31, respectively.

**Fig. 32:**
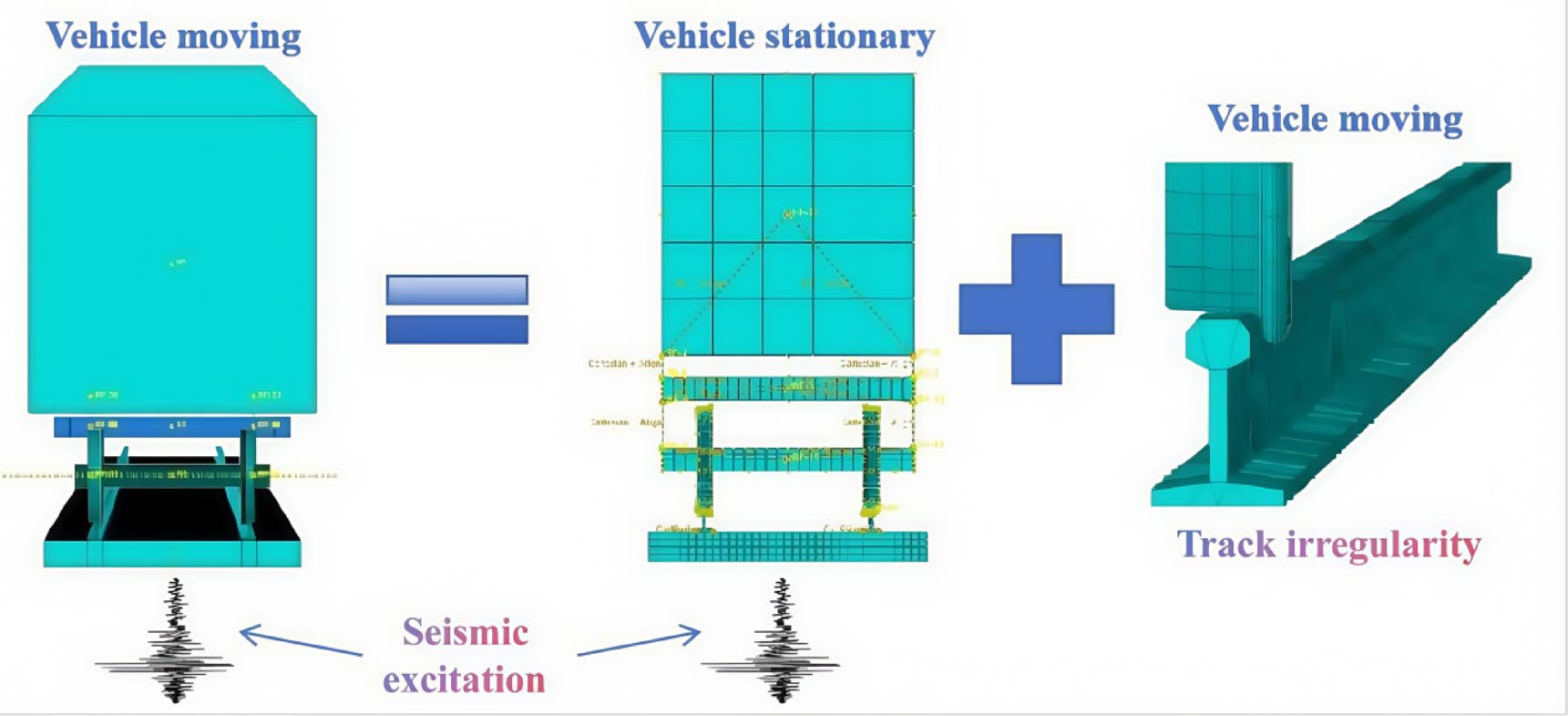
Vehicle-track earthquake response superposition principle (Fig. 32 were generated using^51^).

As can be seen from Figs. 25 and 26, the derailment coefficient for “State B” ranges from 0 to 0.32, and the wheel load reduction rate for “State B” ranges from 0 to 0.34. From Figs. 28 and 29, it is observed that the derailment coefficient for “State C” ranges from 0 to 0.16, and the wheel load reduction rate for “State C” ranges from 0 to 0.33. Figures 31 and 32 indicate that for “State A,” the derailment coefficient ranges from 0 to 0.58, and the wheel load reduction rate ranges from 0 to 0.65.

The fluctuation ranges of the derailment coefficients and wheel load reduction rates for the three states, as well as the fluctuation ranges of the derailment coefficients and wheel load reduction rates under different vehicle speeds for State A, are summarized in Table 6. It is observed that when the vehicle is in State A, although an increase in vehicle speed has some impact on the derailment coefficient and wheel load reduction rate, the fluctuation ranges of the derailment coefficients for the three states generally conform to the pattern “State A” = “State B” + “State C”. Moreover, according to the calculation method of the wheel load reduction rate, its maximum value is 1, hence the fluctuation ranges of the wheel load reduction rates for the three states also approximately conform to the pattern “State A” = “State B” + “State C”. Therefore, it can be concluded that the derailment indicators of vehicles under seismic action can be obtained by the superposition of track irregularities and seismic action. Furthermore, the operating state of vehicles under seismic action can be considered as a superposition of vehicles operating on irregular tracks and vehicles operating on straight tracks under seismic action, with the latter being approximately equivalent to the state of vehicles being stationary on the track under seismic action, i.e., the vehicle-track vibration table test. Therefore, the operating state of vehicles under seismic action can be simplified as a superposition of vehicles operating on irregular tracks and vehicles being stationary on the track under seismic action, as shown in the Fig. 32.

**Table 6:** Three states of derailment coefficient, wheel weight reduction rate fluctuation range.

State	State description	Speed (km/h)	Derailment coefficient	Wheel load reduction rate
C	Earthquake load effect	0	0–0.33	0–0.85
B	Track irregularity excitation effect	300	0–0.32	0–0.29
A	Combined effect of earthquake and track irregularity	100	0–0.76	0–0.64
		200	0–0.85	0–0.77
		300	0–0.92	0–0.99

## Research on earthquake early warning thresholds for high-speed railways

### Preliminary approach to determining earthquake early warning thresholds

In the process of railway design, construction, and maintenance, indicators such as the derailment coefficient and wheel load reduction rate are commonly used to assess the safety of trains under normal operating conditions. According to maintenance standards, there is a certain safety margin between the limit values of the derailment coefficient and wheel load reduction rate and the extreme state of derailment. For instance, the maintenance limit for the derailment coefficient is set at 0.8, while the International Union of Railways (UIC) Code stipulates a derailment coefficient limit of Q/P ≤ 1.2. This indicates that the derailment coefficient of a train under normal operating conditions has a considerable safety margin.

By conducting a comparative analysis of the train derailment indicators under track irregularities, seismic action, and their combined effects, the derailment patterns of operating trains under seismic conditions are revealed. The results show that under seismic action, the derailment indicators of a train can be determined by the superposition of two states: track irregularities and seismic action.

Based on the aforementioned analysis, it is possible to utilize the safety redundancy of train operating states and the principle of superposition as a way to determine the earthquake early warning thresholds. The advantage of this approach is that the dynamic response of the train-track system in a stationary state under seismic action can be verified through shaking table tests. However, it should be noted that the actual operation of trains and seismic action may produce coupling effects.

The computational results presented in “Analysis of wheel-rail dynamic response considering track irregularity” section of the thesis indicate that when considering track irregularities under seismic action, an increase in train speed within a certain range does have some impact on the peak response of derailment indicators, but the impact is limited. Therefore, the aforementioned approach can be used as a preliminary approximation for determining the earthquake early warning thresholds for high-speed railways.

This paper, based on numerical simulation, will propose preliminary suggestions for earthquake early warning thresholds, which include:

1. The operating state of the train under seismic action is considered as a superposition of two states: first, the train running on an irregular track, and second, the train running on a smooth track under seismic action. The wheel-rail forces when the train is running on a smooth straight track can be approximated as equivalent to the wheel-rail forces when the train is stationary (ignoring random wheel-rail creep forces). Based on this, we can use the static method of the train under seismic action to calculate derailment indicators such as the derailment coefficient and wheel load reduction rate.
2. According to China’s “High-Speed Railway Design Specification (Trial)” ^48^, the limit value of the train’s derailment coefficient is set at Q/P ≤ 0.8, which means the dynamic safety limit for high-speed train operation is 0.8. This value can serve as the safety limit for high-speed trains running on irregular tracks. At the same time, the International Union of Railways (UIC) Code stipulates a derailment coefficient limit of Q/P ≤ 1.2, which means there is a safety margin of 0.4. According to the superposition principle in item (1), and allowing for a certain safety margin, it is reasonable to take half of the safety margin, that is, 0.2, as the limit value of the derailment coefficient for the train in a static state under seismic action.
3. Using typical seismic records, determine the relationship between the derailment coefficient and other parameters of the stationary train under seismic action and the peak ground acceleration through numerical simulation, and then determine the earthquake early warning threshold for high-speed railway trains based on the limit value of the derailment coefficient.

### Determination of earthquake early warning thresholds

To study the earthquake early warning thresholds for trains, the two-dimensional vehicle-track dynamics model described earlier is employed. A dynamic response analysis is conducted on the vehicle-track model using six typical earthquake waves. The details of these six earthquake waves are as follows: Taiwan Chi-Chi Earthquake Wave, with a duration of 30 s and a peak acceleration of − 357.5 cm/s^2^. California El-Centro Earthquake Wave, with a duration of 30 s and a peak acceleration of 334.8 cm/s^2^. Japan Kobe Earthquake Wave, with a duration of 25 s and a peak acceleration of 337.9 cm/s^2^. Tianjin Earthquake Wave, with a duration of 19.19 s and a peak acceleration of − 104 cm/s^2^. Wenchuan Wolong Earthquake Wave, with a duration of 30 s and a peak acceleration of 653 cm/s^2^. United States Northridge Earthquake Wave, with a duration of 30 s and a peak acceleration of 556.9 cm/s^2^.

These earthquake waves are selected to cover a range of seismic activities that could provide insights into the dynamic behavior of the vehicle-track system under different levels of ground motion. The analysis will help in understanding how the train and track system responds to various seismic events and contribute to establishing appropriate early warning thresholds for earthquake scenarios.

**Fig. 33:**
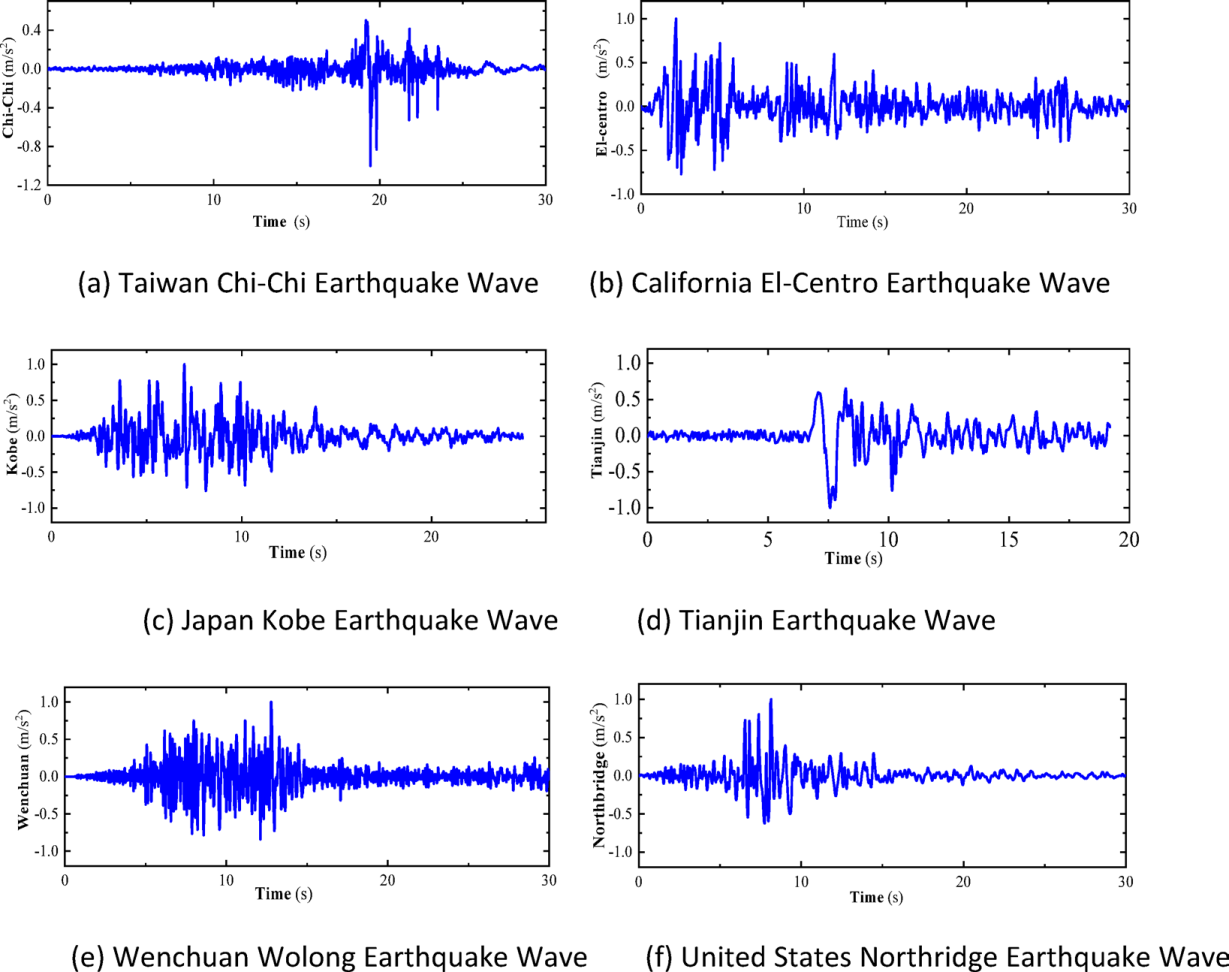
Seismic wave acceleration peak 0.1 g time history curve.

**Fig. 34:**
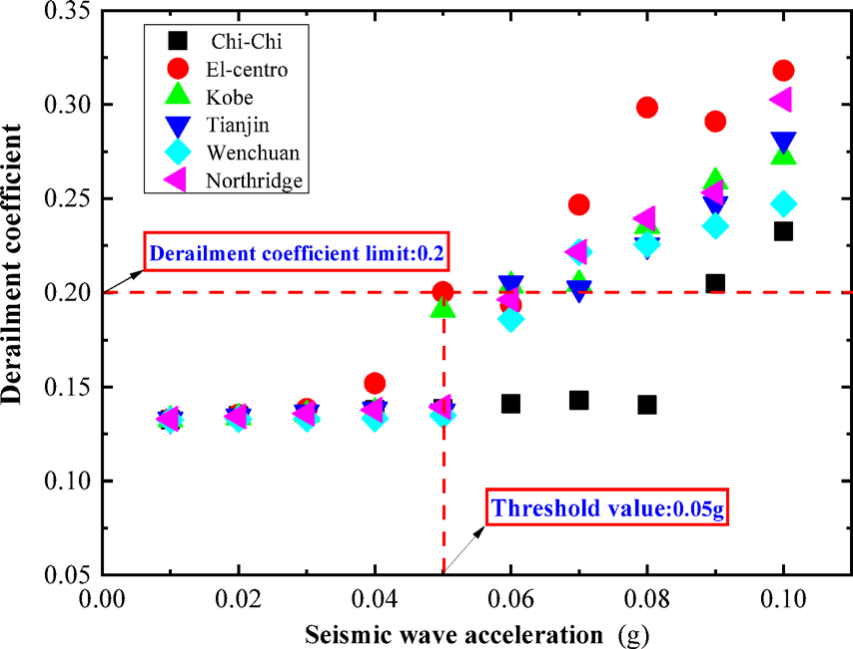
Relationship between derailment coefficient and peak acceleration of seismic wave.

Normalized processing is applied to the accelerations of the six earthquake waves, adjusting the peak accelerations of each earthquake wave in increments of 0.01 g to values of 0.01 g, 0.02 g, and so on, up to 0.1 g. Figure 33 shows the acceleration time-history curves when the peak acceleration of each earthquake wave is set to 0.1 g. These standardized earthquake waves are then sequentially input at the base of the vehicle-track model for time-history analysis calculations. The model’s derailment coefficients and wheel load reduction rates under the influence of each level of earthquake wave are calculated. Curves are plotted with the peak acceleration values of each level of earthquake wave as the horizontal axis and the maximum response values of the derailment coefficients under the influence of each level of earthquake wave as the vertical axis, as shown in Fig. 34.

As can be seen from Fig. 34, under the action of an earthquake, when the train is in a stationary state, the limit value of the derailment coefficient is set at 0.2. After comparing the effects of six different earthquake waves, it was found that only when the peak acceleration of the earthquake wave reaches 0.05 g, as shown by the El-Centro earthquake wave, will the train’s derailment coefficient reach this limit value. This finding provides us with an important reference basis. Based on the above analysis, it is reasonable to set the peak acceleration of 0.05 g as the earthquake early warning threshold for our country’s high-speed railway system. The establishment of this threshold is not only based on detailed data and scientific analysis but also fully considers the safety margin of the train under earthquake conditions, providing a clear reference standard for the earthquake safety of high-speed railways. Through the application of this threshold, high-speed railway managers can quickly assess the risk during an earthquake and take timely early warning measures to ensure the safety of passengers and trains.

## Conclusion

The earthquake disaster prevention and mitigation for high-speed railways mainly involve two levels of issues: First, the seismic safety issues of the high-speed railway’s own infrastructure; second, the safety issues during the train’s operation under earthquake conditions (which is still related to the seismic safety of the high-speed railway infrastructure). For the first issue, the seismic safety of the high-speed railway engineering structure can be addressed through seismic-resistant methods, which play a crucial role in ensuring the seismic safety of the engineering structure. However, in the face of safety issues during the train’s operation under earthquake conditions, more in-depth and effective solutions need to be explored. A possible solution is to set up an earthquake early warning threshold to identify earthquake risks in advance and take corresponding preventive measures. This paper, through shaking table tests, two-dimensional numerical models, and three-dimensional numerical models considering track irregularities, has conducted a comprehensive analysis and calculation, and reached the following main conclusions:

1. Numerical Model Validation: By comparing the numerical simulation results of the two-dimensional vehicle-track model with the shaking table model test results, it was found that the overall trends are basically consistent. Although there are differences in detail, the numerical calculation results are reliable in terms of periodic characteristics and peak responses, verifying the correctness of the two-dimensional vehicle-track numerical model.
2. Impact of Train Speed: Without considering track irregularities, the vertical force between the wheel and the rail, horizontal force, wheel lift, and horizontal relative displacement curves at different train speeds (100 km/h, 200 km/h, 300 km/h) show little difference, indicating that the impact of train speed on wheel-rail dynamic response is limited. In addition, comparing with the shaking table test results of the static vehicle-track model, the dynamic response of the wheel-rail under earthquake action when the train is stationary is similar to that when the train is running at a constant speed on a smooth track, thus the stationary state of the train under earthquake action can be approximated as the state of the train running at a constant speed on a smooth and straight track.
3. Impact of Track Irregularities: Considering track irregularities, with the peak acceleration of the seismic wave at 0.3 g and the wavelength of track irregularities ranging from 0.5 to 80 m, and the train speed ranging from 100 to 300 km/h, the train speed has a certain impact on the peak response of various derailment indicators, but the impact is not significant, indicating that under certain conditions, the risk of derailment under earthquake action is not greatly related to vehicle speed.
4. Analysis of Derailment Patterns: By comparing the impact of track irregularities, earthquake action, and their combined effect on train derailment indicators, the derailment patterns of operating trains under earthquake action are revealed. The study shows that the derailment indicators of trains under earthquake action can be determined by the superimposed effect of track irregularities and earthquake action, thus the operating state of the train under earthquake action can be viewed as a superposition of running on an irregular track and running on a smooth and straight track under earthquake action.
5. Suggestion for Earthquake Early Warning Threshold: Through the calculation and analysis of six different horizontal seismic waves (Jiji wave from Taiwan, El-Centro wave, Kobe wave, Tianjin wave, Wenchuan Wolong wave, Northridge wave) on the two-dimensional vehicle-track model, combined with the safety margin and superposition principle of derailment indicators, it is suggested that the different derailment coefficient limits for the train in a static state under earthquake action be set at 0.2, and the corresponding earthquake early warning threshold for high-speed railways is recommended to be set at 50 gal.

## Data Availability

Data will be made available upon reasonable request by contacting the corresponding author.
